# Comment on Marsigliante et al. Effects on Children’s Physical and Mental Well-Being of a Physical-Activity-Based School Intervention Program: A Randomized Study. *Int. J. Environ. Res. Public Health* 2023, *20*, 1927

**DOI:** 10.3390/ijerph20237131

**Published:** 2023-12-01

**Authors:** Raphiel Murden, Jon Agley, Lilian Golzarri-Arroyo, Armando Peña, Danny Valdez, Abu Bakkar Siddique, Moonseong Heo, David B. Allison

**Affiliations:** 1 Department of Biostatistics and Bioinformatics, Rollins School of Public Health, Emory University, Atlanta, GA 30322, USA; 2 Department of Applied Health Science, School of Public Health-Bloomington, Indiana University, Indianapolis, IN 46805, USA; jagley@indiana.edu (J.A.);; 3 Biostatistics Consulting Center, School of Public Health-Bloomington, Indiana University, Indianapolis, IN 46805, USA; 4 Department of Health and Wellness Design, School of Public Health-Bloomington, Indiana University, Indianapolis, IN 46805, USA; 5 School of Public Administration, Florida Atlantic University, 777 Glades Road, Boca Raton, FL 33431, USA; 6 Department of Public Health Sciences, College of Behavioral, Social and Health Sciences, Clemson University, Clemson, SC 29634, USA; 7 Department of Epidemiology and Biostatistics, School of Public Health-Bloomington, Indiana University Bloomington, Bloomington, IN 47405, USA

**Keywords:** physical activity, pediatric well-being, randomized clinical trials, clustered randomized trials

## Abstract

We conducted a critical review of the article “Effects on Children’s Physical and Mental Well-Being of a Physical-Activity-Based School Intervention Program: A Randomized Study”, published in the *International Journal of Environmental Research and Public Health* in 2023 as part of the Special Issue “Psychomotricity and Physical Education in School Health”. We identified multiple mistakes in the statistical analyses applied. First, the authors claim to have found a statistically significant association between the proposed intervention and change in body composition (body mass index (BMI) percentiles, relative fat mass, and BMI classes) by way of exhibiting differences in nominal significance between the pre- and post-intervention changes *within* the control and intervention groups, instead of exhibiting a significant difference *between* groups. Furthermore, the analysis described fails to account for clustering and nesting in the data. The reporting of the statistical methods and results include multiple elements that are variously incorrect, incoherent, or impossible. Revised statistical analyses are proposed which can render the study’s methods valid and its results substantiated, whereas the current methods and results are invalid and unsubstantiated, respectively.

## 1. Introduction

The study conducted in [1] is described as a randomized clinical trial designed to examine the effectiveness of a novel intervention of physical activity on several measures of body composition, cognition, and overall physiological well-being (PWB) in a pediatric population. The article claims to show that participants who underwent a 6-month intervention exhibited marked improvement in each of these three domains as compared to the participants in the control arm of the study. The investigators also claim that the differences between the control and intervention groups were independent of the participants’ sex. The data analyzed were from a sample of 310 children (139 boys vs. 171 girls) between the ages of 8 and 10 years, randomized to either the intervention or control group by classroom. That is, randomization occurred at the classroom level. Each child was in one of fourteen 4th- or 5th-grade classes at five different schools within three cities in the province of Lecce, Italy. While this is an innovative, important study, we identified several unequivocal errors in the statistical analyses and presentation of the results, and here, we offer solutions for correction. The errors are as follows: (1) the central claim of a reduction in body mass index (BMI) is based on a difference in the nominal significance (DINS) error as defined in [2]; (2) the data are analyzed as if they originate from a randomized clinical trial, whereas the study design reflects a cluster randomized trial (CRCT), as described in [2]; (3) the results are reported with stratification by sex, but no such analyses are described in the ‘Methods’ section; and (4) the ‘Results’ section contains several misinterpretations or possible typographical errors. We offer solutions for each of these points with the goal of ensuring that the study’s reported outcomes are substantiated by appropriate analyses and interpretations, which, at present, they are not.

The remainder of this article is organized into the following sections: 2–5 outline each of the four issues mentioned above and offers solutions to resolve them, respectively, and Section 6 provides a short discussion.

## 2. Difference in Nominal Significance

Aside from examining for baseline differences, the first result reported in [1] describes a decrease in BMI in the intervention group compared to the control group. Supporting evidence for this claim is given as “*p* < 0.01 by Student’s *t*-test, Figure 2A”. The referenced figure displays distributions of the BMI percentiles in each study arm at each time-point (baseline = T0 and 6-month = T1), stratified by sex. However, it is unclear what variables were used in the hypothesis test supporting this statement. The figure and its caption indicate *p*-values from paired *t*-tests, which are inconsistent with the *p*-value given in the text and appear to arise from tests of the data stratified by sex.

An appropriate analysis for testing the hypotheses about whether changes in body composition measures (e.g., BMI) were associated with the study intervention should examine differences between groups rather than describe how the changes within groups differed [3]. For example, assuming *arguendo* (although it is not actually true) that the nested clustering of the students inside the classrooms inside schools and gender did not exist, a two-sample *t*-test of the change in body composition measures would be more appropriate. An alternative method that could also take sex into account would be to use a linear model where the body composition measure at follow-up is the outcome variable and the baseline measure is an explanatory variable.

## 3. Clustering in Randomization Scheme

The study is described a randomized clinical trial [4], but the randomization scheme given in Section 2.2 of [1] describes a CRCT, as described in [2,5,6,7], because entire classrooms, rather than individuals in classrooms, were randomized to the intervention or control arm of the study. When such randomization occurs, the variation in the outcome can be attributed to variance between clusters and variance within clusters, which can result in both inflated type I and type II errors [2,6].

For the proposed hypothesis tests, linear mixed-effects models with random intercepts for the classrooms and adjustment of the degrees of freedom for the number of clusters, instead of subjects, would have been one appropriate approach [8]. It would yield an estimate of the treatment effect that is analogous to a treatment effect observed in a linear model and can be implemented on most statistical software platforms.

## 4. Results Stratified by Sex

The statistical analysis section in [1] states that “…paired and independent Student’s *t*-tests were used and within-group and between-group differences were evaluated, respectively. A two-way repeated measures ANOVA was used when the subjects had undergone two or more conditions”. However, many of the results presented are stratified by sex, with indicators of *p*-values that differ between the sexes. No method accounting for sex is described in the statistical analysis section of the article.

Insufficient detail in describing the statistical methods used constitutes an error in reporting that can limit readers’ ability to assess the validity of the results, preclude reproducibility, and prevent inclusion in meta-analyses. A revised article should include additional details about which methods were used and how potential confounders, such as sex, were accounted for. Including syntax from the statistical software within the supplemental or online materials would alleviate this problem and leave no room for ambiguity.

## 5. Inappropriate Presentation of Results

The first paragraph of the results section in [1] examines differences in the baseline measures between the control and intervention groups and between sexes. It includes the statement “…there were no significant differences between the [control and intervention] groups as regards the proportion of overweight children or children with obesity (*p* > 0.05, by Student’s *t* test; Table 2)”. However, Table 2 does not exhibit proportions of children with overweight or obesity, and a Student’s *t*-test would be inappropriate to test whether these proportions differed across groups.

In the second paragraph on page 7 of [1], which describes the results of the two-factor repeated-measures ANOVA, the same F-statistic and degrees of freedom are listed three times, with three different *p*-values and three different effect sizes. Such results are mathematically impossible. Perhaps the reader may assume that the F-statistic listed describes the overall model fit for a model that includes an interaction between sex, group, and time. In this case, the relevant coefficients or statistics should have been presented because the surrounding text and *p*-values seem to refer to explanatory variables. On the other hand, if the *p*-values and effect sizes come from different models, then the F-statistics should differ in value.

In presenting the analyses of the BMI classes, the investigators claim that differences were observed in the proportions of BMI classes between the control and intervention groups and before and after the intervention using Fisher’s exact test. The figure’s caption suggests that the analyses were stratified by sex. However, it is unclear which two of the three remaining variables depicted (i.e., time, treatment arm, and BMI class) were used to conduct the exact test.

Overall, the presentation of the results was inconsistent and, in places, difficult for multiple readers in our team to interpret. Test statistics of the same value, with the same degrees of freedom and an alternative hypothesis, cannot produce different *p*-values. In parts of the results section, the authors provide some indications regarding how their analyses were conducted. However, the authors should provide a thorough description of which statistical methods were used in the statistical methods section of the article.

Lastly, values should also be checked for implausibility before publication. For example, the standard deviation of the total number of correct responses to the *d*2 test given to girls at T1 differs wildly from similar values in the same table (Table 4). It is plausible that this corresponds to a typographical error. These issues reinforce the necessity of a thorough review and reproduction of all data, analyses, and results before final submission for publication.

## 6. Discussion

Conducting appropriate statistical analyses and clearly presenting the associated results are paramount to advancing scientific integrity. Applying inappropriate statistical methods or incorrectly interpreting the results of appropriately applied statistical methods can lead to incorrect conclusions. When such issues go uncorrected, they can propagate through scientific and broader communities, as others depend on them to inform further research or make decisions [9,10]. Unfortunately, the publishing of such mistakes is not uncommon in the larger scientific community [2].

In the case of [1], the investigators conducted a CRCT, where the clusters were intuitive for the research setting. However, clustering is not accounted for in the statistical analyses. For most of the results presented, the authors discuss whether the effects differ across sex groups. However, there is no mention of how sex was adjusted for in the statistical analyses. Several parts of the article’s ‘Results’ section seem to need editing to ensure that the results are presented clearly and accurately. Lastly, the investigators base several claims regarding the intervention effects on differences in nominal significance rather than differences between groups.

We propose appropriate statistical analyses of the data collected for this study. All analyses should take clustering and nesting into account and should examine differences between the control and intervention groups directly in the statistical analyses, instead of describing differences in the statistical results between groups. There should also be a direct correspondence between the methods that are described and the results that are presented. We request an opportunity to collaborate with the investigators to improve the analyses of these important data and assist them in interpreting their results. Unfortunately, according to the data availability statement, the data from [1] are “unavailable due to privacy”. De-identifying these data on the individual, school, and city levels would mitigate privacy issues and increase transparency, as well as reproducibility.

## References

[1] Marsigliante S., Gómez-López M., Muscella A. (2023). Effects on Children’s Physical and Mental Well-Being of a Physical-Activity-Based School Intervention Program: A Randomized Study. Int. J. Environ. Res. Public Health.

[2] George B.J., Beasley T.M., Brown A.W., Dawson J., Dimova R., Divers J., Goldsby T.U., Heo M., Kaiser K.A., Keith S.W. (2016). Common Scientific and Statistical Errors in Obesity Research. Obesity.

[3] Dimova R.B., Allison D.B. (2016). Inappropriate statistical method in a parallel-group randomized controlled trial results in unsubstantiated conclusions. Nutr. J..

[4] Boutron I., Dutton S., Ravaud P., Altman D.G. (2010). Reporting and Interpretation of Randomized Controlled Trials with Statistically Nonsignificant Results for Primary Outcomes. JAMA.

[5] Campbell M.J., Donner A., Klar N. (2007). Developments in cluster randomized trials and Statistics in Medicine. Stat. Med..

[6] Feng Z., Diehr P., Peterson A., McLerran D. (2001). Selected Statistical Issues in Group Randomized Trials. Annu. Rev. Public Health.

[7] Puffer S., Torgerson D.J., Watson J. (2005). Cluster randomized controlled trials. J. Eval. Clin. Pract..

[8] Murray D.M., Varnell S.P., Blitstein J.L. (2004). Design and analysis of group-randomized trials: A review of recent methodological developments. Am. J. Public Health.

[9] Soumerai S., Koppel R. (2017). How bad science can lead to bad science journalism—And bad policy. The Washington Post.

[10] Zeidler D.L., Sadler T.D., Berson M.J., Fogelman A.L. (2002). Bad science and its social implications. The Educational Forum.

